# Type I Interferon Pathway-Related Hub Genes as a Potential Therapeutic Target for SARS-CoV-2 Omicron Variant-Induced Symptoms

**DOI:** 10.3390/microorganisms11082101

**Published:** 2023-08-17

**Authors:** Zhiwei Lin, Mingshan Xue, Ziman Wu, Ze Liu, Qianyue Yang, Jiaqing Hu, Jiacong Peng, Lin Yu, Baoqing Sun

**Affiliations:** 1 Department of Clinical Laboratory, National Center for Respiratory Medicine, National Clinical Research Center for Respiratory Disease, State Key Laboratory of Respiratory Disease, Guangzhou Institute of Respiratory Health, The First Affiliated Hospital of Guangzhou Medical University, Guangzhou 510120, China; veelin0419@stu.gzhmu.edu.cn (Z.L.);; 2 Guangzhou Laboratory, Guangzhou 510005, China

**Keywords:** COVID-19, SARS-CoV-2, Omicron, biomarker, type I interferon

## Abstract

Background: The global pandemic of COVID-19 is caused by the rapidly evolving severe acute respiratory syndrome coronavirus 2 (SARS-CoV-2). The clinical presentation of SARS-CoV-2 Omicron variant infection varies from asymptomatic to severe disease with diverse symptoms. However, the underlying mechanisms responsible for these symptoms remain incompletely understood. Methods: Transcriptome datasets from peripheral blood mononuclear cells (PBMCs) of COVID-19 patients infected with the Omicron variant and healthy volunteers were obtained from public databases. A comprehensive bioinformatics analysis was performed to identify hub genes associated with the Omicron variant. Hub genes were validated using quantitative RT-qPCR and clinical data. DSigDB database predicted potential therapeutic agents. Results: Seven hub genes (IFI44, IFI44L, MX1, OAS3, USP18, IFI27, and ISG15) were potential biomarkers for Omicron infection’s symptomatic diagnosis and treatment. Type I interferon-related hub genes regulated Omicron-induced symptoms, which is supported by independent datasets and RT-qPCR validation. Immune cell analysis showed elevated monocytes and reduced lymphocytes in COVID-19 patients, which is consistent with retrospective clinical data. Additionally, ten potential therapeutic agents were screened for COVID-19 treatment, targeting the hub genes. Conclusions: This study provides insights into the mechanisms underlying type I interferon-related pathways in the development and recovery of COVID-19 symptoms during Omicron infection. Seven hub genes were identified as promising biological biomarkers for diagnosing and treating Omicron infection. The identified biomarkers and potential therapeutic agent offer valuable implications for Omicron’s clinical manifestations and treatment strategies.

## 1. Introduction

The COVID-19 pandemic, caused by the severe acute respiratory syndrome coronavirus 2 (SARS-CoV-2), has presented an unparalleled global public health challenge. SARS-CoV-2 infection leads to a spectrum of clinical outcomes, ranging from asymptomatic cases to severe illness characterized by symptoms such as high fever, cough, fatigue, and dyspnea, ultimately leading to respiratory failure [1,2]. Since 2019, there have been more than 755 million cumulative cases of COVID-19 globally and more than 6.83 million deaths [3]. No specific antiviral therapy for the pandemic COVID-19 exists yet, particularly for the milder-seeming Omicron variant. Numerous vaccines are under development, and several previously FDA-approved drugs have been repurposed to slow the progression of COVID-19 [4].

Recent real-world studies indicate that the Omicron variant, which is currently predominant, may exhibit milder clinical manifestations compared to earlier variants, with lower hospitalization rates and shorter lengths of stay observed in certain regions [5,6,7,8]. The formation of multinucleated syncytia, reflecting cell–cell fusion during viral infection, represents a crucial pathological step in SARS-CoV-2 infection [9,10]. In vitro, assays have demonstrated the reduced formation of multinucleated syncytia by the Omicron variant compared to previous variants, along with higher cell viability [11,12,13]. This evidence supports the notion that the Omicron variant manifests with reduced severity compared to its predecessors. However, it is crucial to recognize that thousands of deaths continue to occur worldwide, particularly in developing countries where underreporting is common due to factors like limited testing capacity [14]. Furthermore, the highly transmissible Omicron variant continues to strain healthcare systems significantly [15,16]. Therefore, gaining a comprehensive understanding of the underlying pathogenesis of the SARS-CoV-2 Omicron variant and its association with symptomatic presentation remains a paramount research priority.

Type I interferons (IFN-I), including IFN-α and IFN-β, play a pivotal role in the pathogenesis of COVID-19, acting as crucial antiviral factors [17]. Upon activation of the JAK-STAT pathway by the IFN-I receptor complex, the inhibitory effect of type I interferon on SARS-CoV replication suggests its potential for viral clearance [18]. The timing of the IFN-I response varies, with an early response observed in mild cases and a delayed response in severe cases of COVID-19 [19,20]. Single-cell RNA analysis of peripheral blood mononuclear cells from severe COVID-19 patients has revealed up-regulation of IFN-I and other inflammatory cytokines [21]. Thus, the IFN-I pathway may contribute to symptom development, and early administration of IFN-I could potentially enhance viral clearance. A meta-analysis has supported the potential of JAK inhibitor baricitinib as a candidate drug against COVID-19 [22]. The current research on COVID-19 has extensively covered various variants’ clinical manifestations and transmission dynamics, including the Omicron strain [23,24]. However, despite the prevalence of symptomatic infections caused by the Omicron variant, there still exists a gap in understanding the specific molecular mechanisms driving symptom development and potential therapeutic interventions. Unraveling this association could unveil potential therapeutic targets for Omicron-dominant COVID-19 cases [25,26,27].

Addressing existing gaps in the literature, this study employed various methods to investigate the uniqueness of COVID-19 infection with the Omicron variant. These methods included the analysis of gene expression datasets [28], functional annotation using bioinformatics tools, pathway analysis, and construction of protein–protein interaction networks [29,30,31,32]. The obtained results were validated using clinical samples. By applying a bioinformatics approach, this study is the first to identify differences in hub genes and pathways associated with COVID-19 symptoms. Given the heterogeneity of COVID-19 symptoms and the ongoing uncertainty regarding its pathogenesis, our findings hold significant value in contributing to the diagnosis and prognosis of COVID-19.

## 2. Materials and Methods

### 2.1. Data Collection and Differential Expression Analysis of Genes

Microarray gene expression datasets were acquired from the GEO database (accessible at https://www.ncbi.nlm.nih.gov/gds, accessed on 10 January 2023) for COVID-19 patients and their matched controls. The search in the GEO database utilized the keywords “COVID-19”, “symptoms”, “SARS-CoV-2”, and “Omicron”. The selection of the COVID-19 microarray dataset followed specific criteria, including human PBMC samples, mRNA gene expression profiles, a minimum of three samples per group, and array-based expression profiling as the study type. Three datasets, namely GSE201530, GSE179627, and GSE167930, were identified and included in this study.

**Figure 1:**
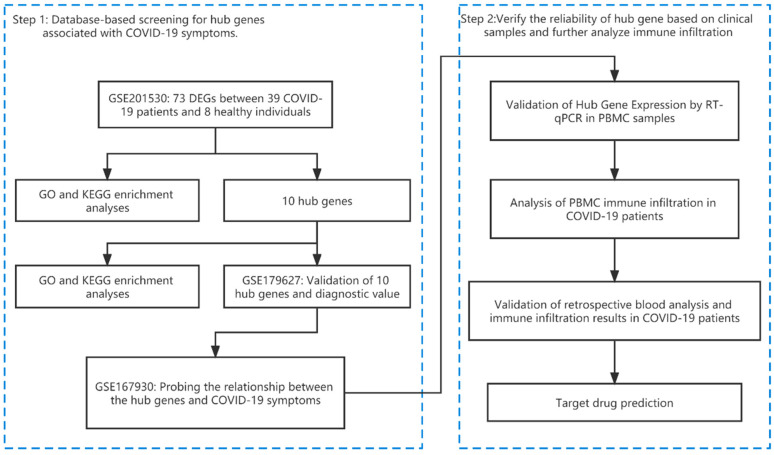
A visual representation of the study design.

The GSE201530 dataset, consisting of 39 COVID-19 patients infected with the SARS-CoV-2 Omicron variant and 8 healthy controls, was utilized for the analysis and identification of hub genes. To validate the diagnostic efficacy of the hub genes, the GSE179627 dataset was used as an independent validation set. Furthermore, the GSE167930 dataset, comprising 21 healthy controls, 7 asymptomatic infected patients, 13 symptomatic infected patients, and 15 recovering patients, was utilized to identify central genes associated with COVID-19 symptoms. A schematic representation of the study design can be found in Figure 1.

To ensure the quality of the dataset samples, we utilized the R package “arrayQualityMetrics”. Data standardization was performed using the “affy” or “limma” packages, renowned for their application in linear models for microarray data. The identification of statistically significant differentially expressed genes (DEGs) between COVID-19 and control samples in each dataset was performed using the “limma (version 3.40.6)” package in R. DEGs with adjusted *p*-values < 0.05 and |log2 Fold change (logFC)| > 2 were considered statistically significant. To visualize the results, volcanic and thermal maps were generated using the R packages “ggplot2 (version 3.3.6)” and “heatmap”, respectively. 

### 2.2. Functional Enrichment Analysis

Gene ontology (GO) is widely used for gene annotation, including molecular functions (MF), biological pathways (BP), and cellular components (CC). Kyoto Encyclopedia of Genes and Genomes (KEGG) enrichment analysis provides valuable insights into gene functions and high-level genomic functional information. To gain a comprehensive understanding of the role of target genes, we utilized the “clusterProfiler (version 4.4.4)” package in R for analyzing GO functions and performing KEGG pathway enrichment analysis. The results of the enrichment analysis were visualized using the “ggplot2 (version 3.3.6)” package. Visualizations such as string and bubble plots were generated to depict the joint logFC enrichment analysis of GO and KEGG, utilizing the “ggplot2 (version 3.3.6)” package.

### 2.3. Protein–Protein Interaction (PPI) Analysis of Differentially Expressed Genes and Identification of Hub Genes

The protein–protein interaction (PPI) network of differentially expressed genes was examined using the STRING database (accessible at https://string-db.org/, accessed on 5 March 2023). Hub genes, which exert a significant influence on other genes within the network, were identified based on their centrality scores. The analysis data from STRING were imported into Cytoscape software (version 3.8.1). We designated the top 10 scoring genes as hub genes using the MCC algorithm from Cytoscape’s cytoHubba plugin. Additionally, the co-expression network of DEGs was analyzed using Cytoscape’s MCODE plugin, and the most significant clusters containing hub genes were visualized. Further analysis involved a Venn diagram to determine the intersection of hub genes obtained from these two methods, resulting in the final set of identified hub genes.

### 2.4. Construction of the miRNA-Target Regulatory Network

To predict the miRNAs and TFs associated with the hub genes, we utilized the miRNet database (accessible at https://www.miRNet.ca/, accessed on 22 Apirl 2023). Subsequently, we constructed and visualized regulatory networks involving mRNA–miRNA and mRNA–TF interactions using Cytoscape software (version 3.8.1).

### 2.5. Validation of the Diagnostic Value of Hub Genes

To assess the sensitivity and specificity of the target genes, we conducted ROC curve analysis using HiPlot software (version 0.1.0). Multigene ROC analysis was performed by calculating the predicted probability of multiple genes contributing to the results in each sample based on a binary Logit model. This analysis was conducted using SPSS 25.0 software. The results were quantified as the area under the ROC curve (AUC), considering genes with an AUC > 0.6 as diagnostically significant.

### 2.6. Immune Infiltration Analysis

The proportion of 22 immune cell types in the samples was estimated using “CIBERSORTx” (accessible at https://cibersortx.stanford.edu/, accessed on 20 May 2023). CIBERSORTx is an analytical tool that utilizes gene expression data to provide mixed estimates of the abundance of different immune cell types within a cell population.

### 2.7. Retrospective Analysis of Blood Counts in COVID-19 Patients

Demographic and clinical information of COVID-19 patients, including gender, age, and symptoms, were collected using an electronic case system. Each isolate was cultured and verified. Continuous variables were presented as median (interquartile range [IQR]). Differences in continuous variables between two groups were assessed using the Mann–Whitney Wilcoxon rank-sum test. A *p*-value < 0.05 was considered statistically significant.

### 2.8. Real-Time Quantitative Polymerase Chain Reaction (RT-qPCR) Verification

To validate the findings obtained from the bioinformatics analysis, peripheral blood mononuclear cell (PBMC) samples were collected from 20 COVID-19 patients infected with the Omicron variant and 20 healthy individuals as controls. Total RNA was extracted using TRIzol reagent (Invitrogen, Carlsbad, CA, USA), and approximately 2 μg of total RNA was reverse transcribed using the iScript cDNA Synthesis Kit (Bio-Rad, Hercules, CA, USA). RT-qPCR was performed on a CFX Connect Real-Time PCR detection system (Bio-Rad) using ChamQ SYBR Color qPCR Master Mix (Vazyme, Nanjing, China). The relative mRNA expression was calculated using the 2^−ΔΔCt^ method. The primer sequences used in the experiment are provided in Table 1. Statistical analysis was performed using one-way analysis of variance with SPSS 25.0 software (IBM, Armonk, NY, USA), and statistical significance was defined as a *p*-value < 0.05.

### 2.9. Prediction of Potential Therapeutic Agents

The DSigDB database (available at http://tanlab.ucdenver.edu/DSigDB, accessed on 30 May 2023) was utilized to predict potential therapeutic agents for COVID-19 based on protein–drug interaction data. The thresholds set for selection were FDR < 0.05 and composite score > 5000.

### 2.10. Statistical Analysis

Statistical analyses were performed using GraphPad Prism 9 and R software (version 4.2.2). The data were presented as mean ± standard deviation, and a comparison between groups was conducted using an unpaired Student’s *t*-test. A *p*-value less than 0.05 was considered statistically significant.

## 3. Results

### 3.1. Screening and Functional Enrichment Analysis of Differentially Expressed Genes in PBMC of Omicron Infection

**Figure 2:**
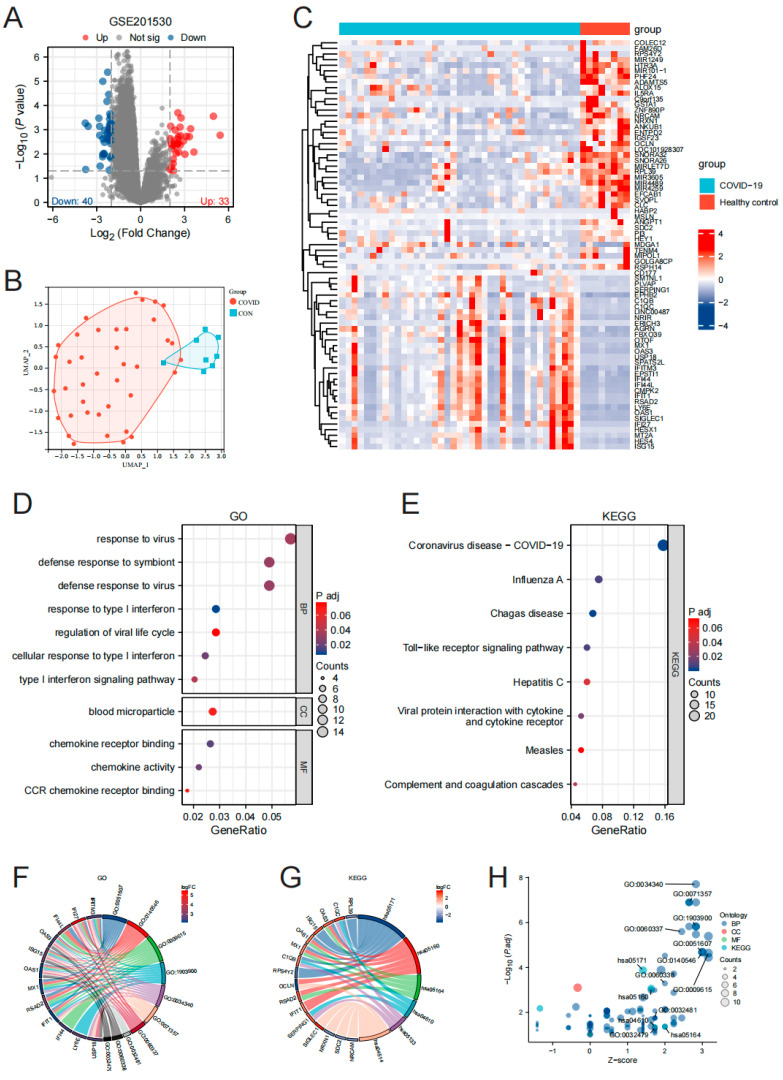
Comprehensive analysis of gene expression and functional annotation in COVID-19. volcano plot (**A**); GSE201530UMAP plot (**B**); heat map of DEGs (**C**); GO/KEGG categories and pathways (**D**,**E**); chord diagram describing the relationship between GO/KEGG terms of genes and biological processes (**F**–**H**).

The differential gene analysis of the GSE201530 dataset was conducted using the “limma” package in R, resulting in the identification of 73 differentially expressed genes (33 up-regulated and 40 down-regulated), as shown in Figure 2A (Supplementary Table S1). The screening criteria applied were |log2(FC)| > 2 and adj. *p*-value < 0.05.

To evaluate the reproducibility of the data within the group, UMAP analysis was performed, demonstrating satisfactory reproducibility, as depicted in Figure 2B. Volcano plots illustrating the differentially expressed genes were generated using the “ggplot2 [3.3.6]” package in R, with the parameters Log2FC > 2 and adj. *p*-value < 0.05, as shown in Figure 2C.

GO and KEGG enrichment analyses were conducted on the differentially expressed genes (Supplementary Table S2). The results revealed significant GO enrichments related to virus response, defense response to symbiont, defense response to virus, response to type I interferon, regulation of viral life cycle, cellular response to type I interferon, and the type I interferon signaling pathway. In the KEGG enrichment analysis, the differentially expressed genes were primarily associated with diseases such as COVID-19, Influenza A, and Chagas disease, as depicted in Figure 2E–H.

### 3.2. Gene Screening and Functional Enrichment Analysis of PBMC Hub Genes in Omicron Infection

**Figure 3:**
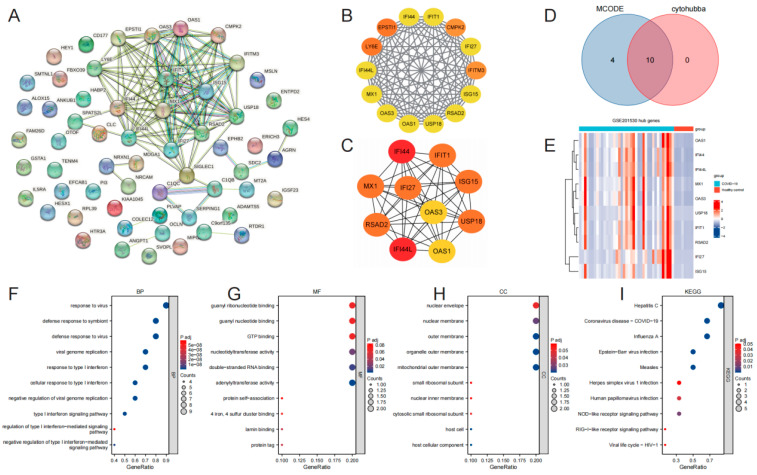
Identification and validation of hub genes. STRING (**A**); MCODE hub genes (**B**); cytohubba-MCC hub genes (**C**); VENN of MCODE hub genes and cytohubba-MCC hub genes (**D**); heatmap of 10 hub genes (**E**); GO/KEGG categories and pathways (**F**–**I**).

The differentially expressed genes obtained earlier were used to construct a protein-protein interaction network using the STRING database (accessible at https://string-db.org/, accessed on 5 March 2023) (Figure 3A). The data from STRING were imported into Cytoscape software (version 3.8.1), and the MCODE plugin was utilized to analyze the co-expression network of the differentially expressed genes. The visualization of the most significant clusters revealed the hub genes: IFI44L, RSAD2, IFI27, MX1, OAS1, LY6E, IFIT1, OAS3, EPSTI1, IFITM3, CMPK2, IFI44, ISG15, and USP18 (Figure 3B). Furthermore, the cytohubba plugin in Cytoscape, employing the MCC algorithm, identified the top 10 scoring genes as hub genes: OAS1, IFI44, IFI44L, MX1, OAS3, USP18, IFIT1, RSAD2, IFI27, and ISG15 (Figure 3C). VENN plots confirmed that these 10 genes exhibited common differential expression in the gene set, thus confirming their status as the final identified hub genes: OAS1, IFI44, IFI44L, MX1, OAS3, USP18, IFIT1, RSAD2, IFI27, and ISG15 (Figure 3D). The visualization of the hub genes was presented in a volcano plot generated using the “ggplot2” package in R software (version 4.2.2) (Figure 3E).

Following the hub genes analysis, GO and KEGG enrichment analyses were performed (Supplementary Table S3). The results demonstrated significant enrichment in GO terms associated with the response to virus, response to type I interferon, cellular response to type I interferon, type I interferon signaling pathway, and regulation of type I interferon-mediated signaling. The KEGG analysis revealed the involvement of multiple viral infectious diseases, including COVID-19 (Figure 3F–I).

### 3.3. Confirmation of Hub Genes Expression and Diagnostic Value in GSE179627

**Figure 4:**
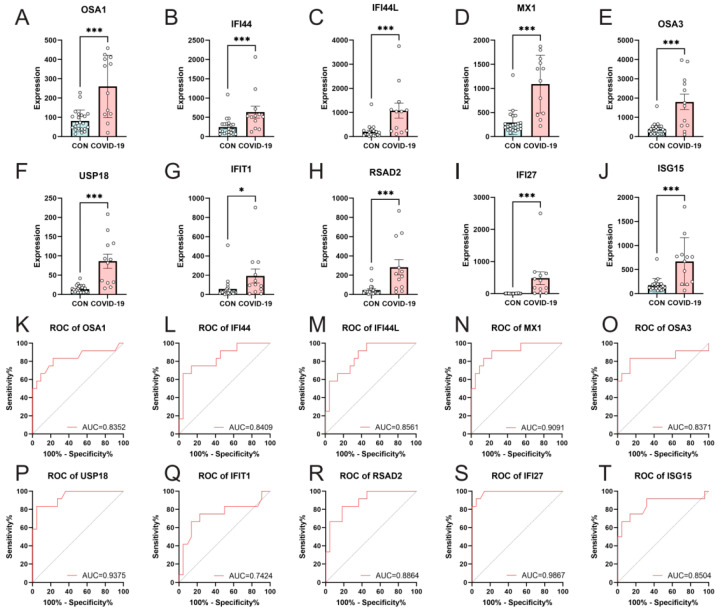
Confirmation of hub genes expression and diagnostic value. Comparison of hub genes expression in the GSE179627 dataset (**A**–**J**); diagnostic ROC curves of COVID-19 versus 10 hub genes in healthy samples (**K**–**T**). *, *p* < 0.05; ***, *p* < 0.001.

GSE179627 was utilized to verify the expression levels of the selected target genes. The results demonstrated consistent expression patterns between COVID-19 patients with Omicron infection and healthy individuals for the 10 hub genes (OAS1, IFI44, IFI44L, MX1, OAS3, USP18, IFIT1, RSAD2, IFI27, and ISG15) (Figure 4A–J).

ROC curves were generated using the data from COVID-19 patients with Omicron infection and healthy individuals to assess the diagnostic value of these 10 genes. The results indicated that these genes hold significant diagnostic value for COVID-19 patients. The AUC values were as follows: OAS1, 0.8352 (95% CI: 0.6697 to 1.000); IFI44, 0.8409 (95% CI: 0.6974 to 0.9844); IFI44L, 0.8561 (95% CI: 0.7306 to 0.9815); MX1, 0.9091 (95% CI: 0.8048 to 1.000); OAS3, 0.8371 (95% CI: 0.6585 to 1.000); USP18, 0.9375 (95% CI: 0.8576 to 1.000); IFIT1, 0.7424 (95% CI: 0.5451 to 0.9397); RSAD2, 0.8864 (95% CI: 0.7744 to 0.9984); IFI27, 0.9867 (95% CI: 0.9591 to 1.000); and ISG15, 0.8504 (95% CI: 0.6896 to 1.000) (Figure 4K–T).

### 3.4. Investigation of the Relationship between Hub Genes and Omicron Infection

**Figure 5:**
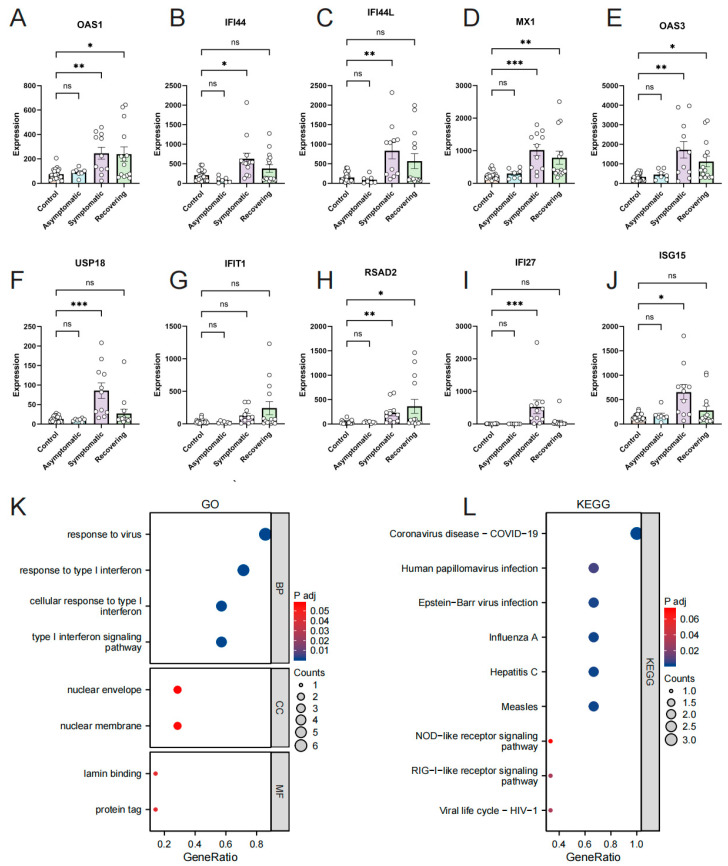
Association of hub genes with COVID-19 symptoms and signaling pathways. Comparison of hub genes expression in different groups (**A**–**J**); GO/KEGG categories and pathways (**K**,**L**). *, *p* < 0.05; **, *p* < 0.01; ***, *p* < 0.001.

To examine the impact of hub genes on the symptoms manifested after SARS-CoV-2 infection in humans, we utilized GSE167930, which included healthy individuals, asymptomatic infected individuals, symptomatic infected individuals, and recovering patients. The analysis revealed no statistically significant difference in the expression of the 10 hub genes between asymptomatic infected individuals and healthy individuals. However, a notable statistically significant difference or a trend towards elevated expression was observed for all 10 hub genes in symptomatic infected individuals compared to both healthy and asymptomatic infected individuals (although the difference was not statistically significant in the latter case). Importantly, during the recovery period of COVID-19, 7 out of the 10 hub genes (IFI44, IFI44L, MX1, OAS3, USP18, IFI27, and ISG15) exhibited a significant decrease, reaching levels comparable to those of healthy individuals (Figure 5A–J).

Furthermore, we conducted GO and KEGG enrichment analyses for the seven hub genes related to COVID-19 symptoms (Supplementary Table S4). The results indicated that the most significant GO enrichment was observed for multiple type I interferon-related categories, including response to virus, response to type I interferon, cellular response to type I interferon, and type I interferon signaling pathway. The KEGG analysis demonstrated the involvement of various viral infectious diseases, including COVID-19. Based on the identified DEGs and the enrichment analyses of the hub genes, the results highlighted the involvement of seven potential biomarkers in abnormal signaling pathways associated with COVID-19 symptom production and recovery, primarily related to type I interferon signaling pathways (Figure 5K,L).

### 3.5. Validation of Hub Genes Expression by RT-qPCR

**Figure 6:**
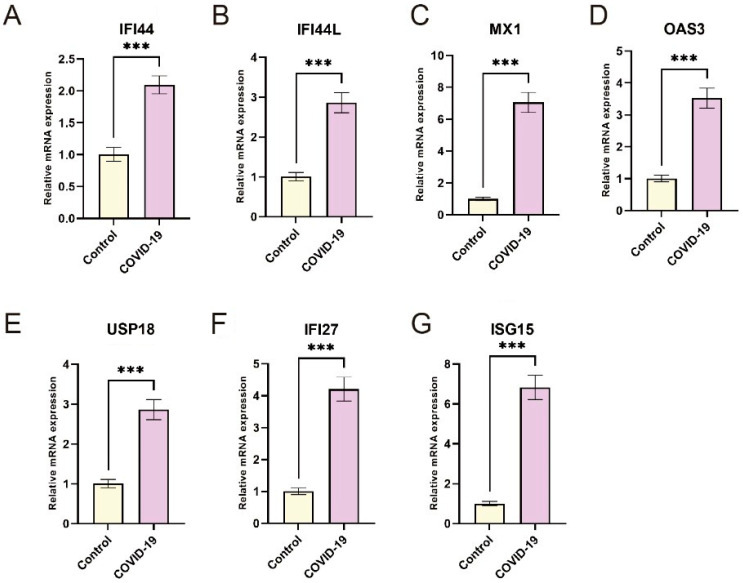
Validation of hub genes expression in COVID-19 patients. RT-PCR analysis of PBMC IFI44, IFI44L, MX1, OAS3, USP18, IFI27, and ISG15 expression from control and COVID-19 (n = 20) (**A**–**G**). ***, *p* < 0.001.

To validate the findings derived from the bioinformatics analysis, PBMC samples were collected from 20 COVID-19 patients infected with the Omicron variant and 20 healthy individuals as controls. The expression levels of the hub genes, namely IFI44, IFI44L, MX1, OAS3, USP18, IFI27, and ISG15, were examined using RT-qPCR. The results revealed a significant upregulation of these genes in the COVID-19 group compared to the control group, which aligns with the patterns observed in the microarray analysis (Figure 6). This validation provides strong support for the reliability of the bioinformatics analysis and reinforces the evidence suggesting the dysregulation of these hub genes in COVID-19.

### 3.6. Construction of mRNA-miRNA and mRNA-TF Regulatory Networks

**Figure 7:**
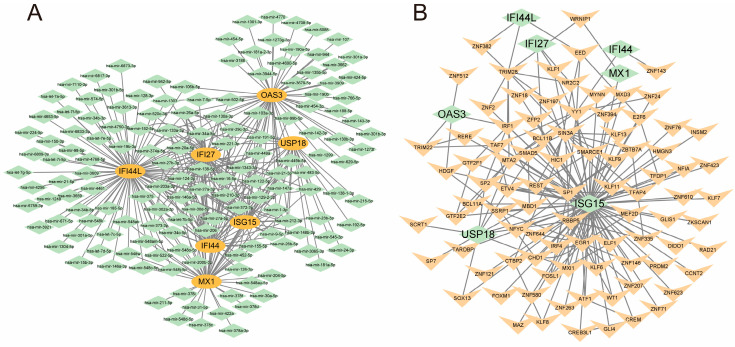
mRNA-miRNA and mRNA-TF interaction networks in COVID-19 symptom-related hub genes. mRNA and miRNAs co-expression network. The network depicts the interaction between the seven hub genes (IFI27, IFI44, IFI44L, ISG15, MX1, OAS3, and USP18) and their corresponding miRNAs. Various miRNAs, such as hsa-mir-146a-5p and hsa-mir-26b-5p, regulate the expression of these hub genes (**A**); mRNA and TF co-expression network. The network shows the interaction between the seven hub genes and transcription factors (TFs). TFs, including NR2C2 and ZNF143, are involved in regulating the expression of these hub genes (**B**).

The miRNet tool was utilized to integrate the results of the seven COVID-19 symptom-related hub genes with the miRNA interaction network, resulting in the identification of 150 miRNAs and 305 mRNA-miRNA pairs. Subsequently, Cytoscape was employed to construct co-expression networks of mRNA and miRNAs (Figure 7A). Notably, there were 63 miRNAs found to regulate IFI27 (e.g., hsa-mir-146a-5p), 36 miRNAs regulating IFI44 (e.g., hsa-mir-26b-5p), 79 miRNAs regulating IFI44L (e.g., hsa-mir-124-3p), 29 miRNAs regulating ISG15 (e.g., hsa-mir-1-3p), 46 miRNAs regulating MX1 (e.g., hsa-mir-204-5p), 57 miRNAs regulating OAS3 (e.g., hsa-mir-143-3p), and 23 miRNAs regulating USP18 (e.g., hsa-mir-26b-5p) (Supplementary Table S5).

Furthermore, the results of the seven COVID-19 symptom-related hub genes were integrated with the transcription factor (TF) interaction network to identify 89 TFs and 179 mRNA-TF pairs. Cytoscape was then employed to construct co-expression networks of mRNA and TFs. The analysis revealed that 3 TFs were involved in regulating IFI27 (e.g., NR2C2), 36 miRNAs regulated IFI44 (e.g., ZNF143) (Figure 7B), 3 TFs regulated IFI44L (e.g., EED), 80 TFs regulated ISG15 (e.g., ZKSCAN1), 1 TF regulated MX1 (WRNIP1), 4 TFs regulated OAS3 (e.g., TRIM22), and 9 TFs regulated USP18 (e.g., MBD1) (Supplementary Table S6).

### 3.7. Analysis of PMBC Immune Infiltration in Omicron Infection

**Figure 8:**
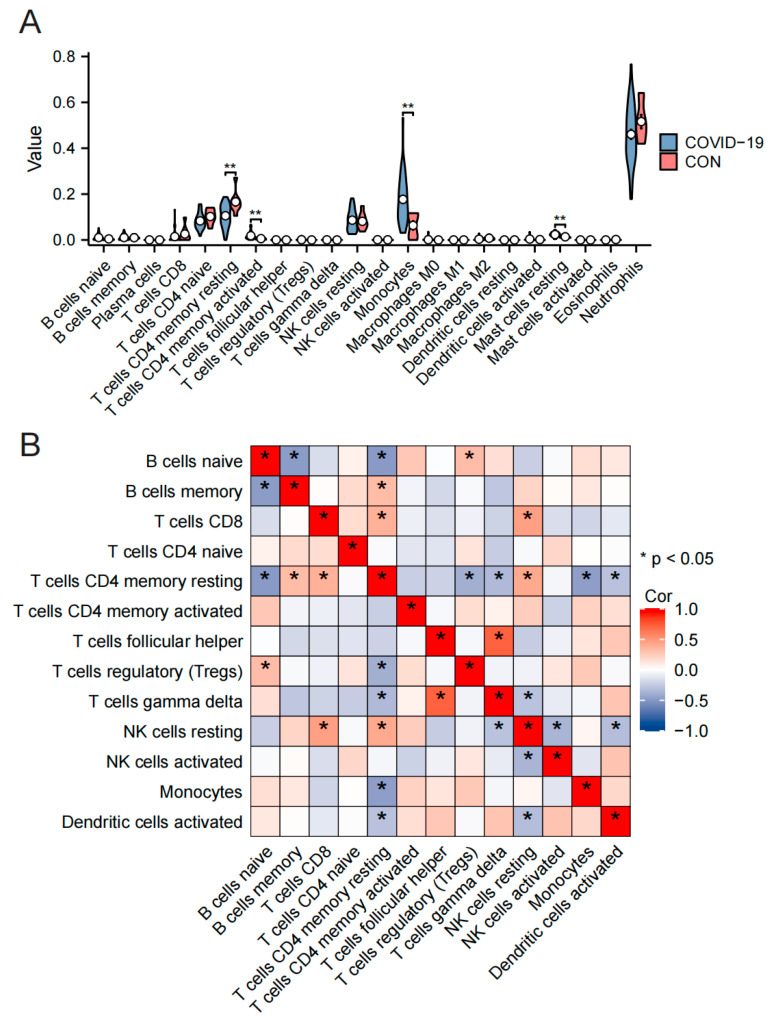
The immune cell infiltration patterns in COVID-19 patients with Omicron infection. Pattern of immune cell infiltration in COVID-19, monocytes, T cells CD4 memory activated, and Mast cells resting ratios were significantly increased, while T cells CD4 memory resting was significantly decreased (**A**). The correlation analysis between immune cells (**B**). *, *p* < 0.05; **, *p* < 0.01.

Based on the GSE201530 dataset, “CIBERSORTx” (https://cibersortx.stanford.edu/, accessed on 20 May 2023) compared the different immune infiltration patterns of COVID-19 patients and normal controls. The results showed that the proportion of monocytes, T cells CD4 memory activated, and Mast cells resting was significantly increased in COVID-19 patients with Omicron infection, while T cells CD4 memory resting was significantly decreased (Figure 8A). Cell types with an expression of 0 that were not present in the sample were further excluded. The PBMC-associated immune cells were selected and the results of correlation analysis between immune cells are shown in Figure 8B.

### 3.8. Validation of Retrospective Blood Analysis and Immune Infiltration Results in Omicron Infection

Neutrophil (NEU%), monocyte (MONO% and MONO), mean corpuscular volume (MCV), mean platelet volume (MPV), platelet large cell ratio (PLCR) and C-reactive protein (CRP) were significantly increased in COVID-19 patients than that in healthy controls (*p* < 0.05) (Table 2). Lymphocytes (LYM and LYM%), eosinophils (EOS and EOS%), basophils (BASO and BASO%), red blood cell (RBC), hemoglobin (HGB), hematocrit (HCT), mean corpuscular hemoglobin concentration (MCHC), blood platelet (PLT), platelet distribution width (PDW), plateletcrit (PCT) and platelet large cell ratio (PLCR) were lower in COVID-19 patients (*p* < 0.05). The elevated monocytes in the COVID-19 group were consistent with the results of the immune infiltration fraction in clinical blood tests. 

### 3.9. Target Drug Prediction

**Figure 9:**
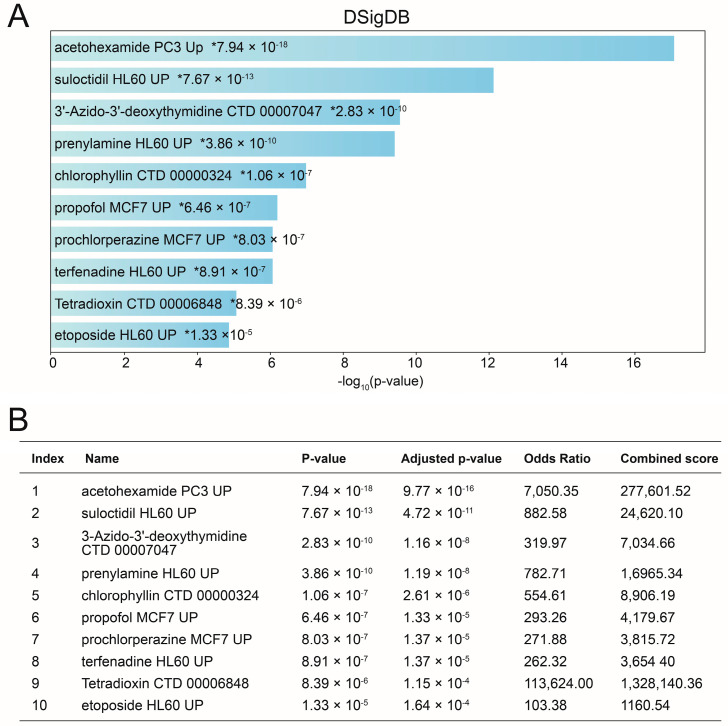
Predicted target drugs for Omicron infection modulating the hub genes. Bar graph of DSigDB (**A**); Table of DSigDB (**B**). The top 10 predicted target drugs, based on composite scores, are presented. Notably, acetohexamide emerges as a promising candidate for the treatment of COVID-19. *, *p*-value.

The DSigDB database was used to predict potential target drugs associated with seve target hub genes that may treat Omicron infection by modulating the hub genes. A total of 123 target drugs were finally predicted; the composite scores and corresponding target genes are listed in Supplementary Table S7. The top 10 predicted target drugs according to the composite scores are shown in Figure 9 The top 10 predicted targets according to the composite score are shown in Figure 9. Among them, acetohexamide is expected to be a potential drug for the treatment of Omicron infection.

## 4. Discussion

The emergence of novel coronaviruses and their potential global impact on public health have become a major concern in recent years [3]. With the ongoing challenges posed by the SARS-CoV-2 Omicron variant, it is crucial to identify potential biomarkers and explore associated mechanisms using bioinformatics approaches to enhance the diagnosis and treatment of this variant.

In this study, we analyzed the PBMC microarray dataset (GSE201530) from the GEO database to identify differentially expressed genes (DEGs) associated with SARS-CoV-2 Omicron variant infection. Through this analysis, we identified 10 hub genes through the construction of a protein–protein interaction (PPI) network. These findings were validated using an independent PBMC dataset of COVID-19 patients (GSE179627), which consistently demonstrated the expected expression patterns of these 10 genes and their diagnostic value. Additionally, we analyzed a dataset comprising healthy individuals, asymptomatic infected individuals, symptomatic infected individuals, and recovered infected individuals (GSE167930) to identify seven target hub genes (IFI44, IFI44L, MX1, OAS3, USP18, IFI27, and ISG15) that showed correlations with symptom onset and recovery from COVID-19. Further GO and KEGG pathway analyses at different stages of genetic screening revealed significant enrichment in pathways related to virus response, defense response to virus, response to type I interferon, cellular response to type I interferon, and the type I interferon signaling pathway. Moreover, we analyzed the miRNAs corresponding to these seven target hub genes and constructed interaction networks with transcription factors (TFs) using the miRNet tool.

In the analysis of the GSE201530 dataset with the “CIBERSORTx” tool, we observed a significant increase in the proportion of monocytes, activated memory CD4 T cells and resting mast cells in COVID-19 patients. Conversely, we observed a notable decrease in the proportion of resting memory T cells CD4. Furthermore, we identified a correlation between elevated monocyte levels and decreased proportions of resting memory T cells CD4. To support these findings, we reviewed blood counts recorded in hospital medical records of symptomatic COVID-19 patients with Omicron infection, which demonstrated that abnormally elevated monocyte ratios and higher monocyte counts were associated with an increased risk of developing symptomatic COVID-19 compared to normal subjects, aligning with the results obtained from immune infiltration analysis.

Moreover, we conducted RT-qPCR to validate the up-regulated expression levels of these seven genes in PBMCs of COVID-19 patients infected with the Omicron variant. The consistent up-regulation of these genes supports their potential crucial role in the progression of COVID-19. The expression trends of many key DEGs found in this study align with previously reported results, further validating the reliability of the database and our data analysis.

Among the seven target hub genes, MX1, OAS3, USP18, IFI27, and ISG15 are characterized as type I interferon-related genes [33]. MX1 acts as an effector protein of the IFN system and plays a crucial role in responding to SARS-CoV-2 infection, with its expression significantly increasing with viral load escalation [34]. Importantly, MX1 directly impacts the viral ribonucleoprotein complex, and its antiviral function relies on the essentiality of its gTPase activity [35]. The interferon-induced antiviral enzyme known as 2′-5′-oligoadenylate synthase (OAS) encompasses OAS1, OAS2, OAS3, and OASL [36]. OAS3 serves as a key player in antiviral action and signal transduction [37]. Additionally, IFI27 (also referred to as ISG12 or p27), an interferon α-inducible gene (and to a lesser extent, interferon γ), exhibits nuclear membrane localization and contributes to diverse biological processes [37]. Notably, a cohort study demonstrated that IFI27 expression was observed in the blood of COVID-19 patients and positively correlated with elevated viral load [38].

IFI44L, an IFN-inducible protein with similarities to IFN-I, is induced by various viruses [39]. As an IFN-I negative regulator, IFI44L mitigates antimicrobial inflammatory factors by negatively regulating the NF-κB pathway and inhibiting STAT1 activation, thereby inhibiting IFN-I and ISG production [40]. Acting as a feedback regulator of the IFN response, IFI44L potentially promotes viral replication by modulating the innate immune response following viral infection [41]. Interestingly, our study reveals a significant overlap between these seven target hub genes and the results obtained from bioinformatics analyses of diseases such as dengue fever [42]. Previous studies have demonstrated shared pathophysiological pathways between dengue fever and COVID-19, including fever, plasma leakage, low platelet count, and coagulation disorders [43]. Therefore, further discussions are warranted to explore potential therapeutic approaches to symptomatic COVID-19 by referencing strategies employed against dengue fever.

The initial defense against viral infection is the interferon I (IFN-I) response, which induces the activation of hundreds of interferon-stimulated genes (ISGs) through the JAK/STAT signaling pathway [44]. Severe lung inflammation leading to respiratory failure in SARS coronavirus type 2-infected patients is primarily attributed to cytokine dysregulation. Severe cases of COVID-19 exhibit impaired production of both IFN-I and interferon II (IFN-II) and downregulation of ISGs [45,46]. The hub genes identified in this study have the potential to counteract COVID-19 development by modulating interferon activity. To identify potential compounds, we conducted a screening process based on the DSigDB database. Among the ranked *p*-values, acetohexamide emerged as a promising candidate for COVID-19 patients with Omicron infection treatment. A study utilizing three molecular docking programs aimed to repurpose FDA-approved drugs targeting the functional structural domain of csBiP, specifically the BiP functional domain, as antivirals against COVID-19, and acetohexamide was among the selected ligands [47]. The potential therapeutic role of acetohexamide in the treatment of COVID-19, particularly in the context of Omicron infection, warrants further investigation and validation through in vitro and clinical studies. Such studies will help elucidate its mechanism of action and assess its safety and efficacy in combating the virus. This research direction represents a promising avenue for the development of targeted therapies to mitigate the impact of COVID-19, especially in the context of emerging variants like the Omicron variant.

Compared to previous studies on Omicron variants, this study brings innovation by specifically focusing on targets associated with symptom development and outcomes. The identification of hub genes related to the Type I interferon pathway highlights their critical role as key therapeutic targets. The validation of our results using patient samples in a clinical setting enhances the accuracy and scientific validity of our findings. However, this study has several limitations that should be acknowledged and taken into consideration. Firstly, there is still a lack of sufficient transcriptomic research related to Omicron variant infection, particularly concerning the differential expression of genes in individuals with different symptoms, leading to a scarcity of available datasets. Secondly, the diagnostic value of research findings such as hub genes and predicted drugs needs to be further verified by additional experimental exploration and clinical trials to establish their potential clinical relevance and utility, which is the direction of future research.

Despite these limitations, this study provides valuable insights into the molecular mechanisms underlying the pathogenesis of the SARS-CoV-2 Omicron variant and its association with the symptomatic presentation. The identified hub genes and enriched pathways offer potential targets for further investigation and the development of therapeutic interventions for Omicron-dominant COVID-19 cases. Further research in this area will be crucial for advancing our understanding of the virus and improving the management of COVID-19 patients.

## 5. Conclusions

In this study, our investigation successfully identified seven hub genes (IFI44, IFI44L, MX1, OAS3, USP18, IFI27, and ISG15) associated with antiviral activity and the type I interferon response as potential biomarkers for the diagnosis and treatment of COVID-19 in individuals infected with the Omicron variant. Through the construction of a comprehensive network of mRNA–miRNA and mRNA–TF interactions, we gained valuable insights into the regulatory mechanisms underlying Omicron infection. Our immune infiltration analysis and retrospective clinical data analysis provided compelling evidence of a correlation between elevated monocytes and the development of Omicron infection. We also identified ten agents as promising drug candidates that specifically target these hub genes for treating Omicron infection. These findings significantly advance our understanding of COVID-19 symptom development and recovery and highlight the potential of further investigating type I interferon-related pathways to identify therapeutic targets and biomarkers for COVID-19 patients, particularly those infected with the Omicron variant.

## Figures and Tables

**Table 1:** Primer sequences used for RT-qPCR.

Gene	Sequence (5′ -> 3′)		Length	Tm	Location
IFI27	Forward Primer	TGCTCTCACCTCATCAGCAGT	21	62.9	12–32
	Reverse Primer	CACAACTCCTCCAATCACAACT	22	60.2	126–105
IFI44	Forward Primer	ATGGCAGTGACAACTCGTTTG	21	61.1	1–21
	Reverse Primer	TCCTGGTAACTCTCTTCTGCATA	23	60	212–190
IFI44L	Forward Primer	AGCCGTCAGGGATGTACTATAAC	23	61	133–155
	Reverse Primer	AGGGAATCATTTGGCTCTGTAGA	23	60.8	248–226
ISG15	Forward Primer	CGCAGATCACCCAGAAGATCG	21	62.6	89–109
	Reverse Primer	TTCGTCGCATTTGTCCACCA	20	62.4	240–221
MX1	Forward Primer	GTTTCCGAAGTGGACATCGCA	21	62.9	7–27
	Reverse Primer	CTGCACAGGTTGTTCTCAGC	20	61.2	128–109
OAS3	Forward Primer	GAAGGAGTTCGTAGAGAAGGCG	22	62.1	66–87
	Reverse Primer	CCCTTGACAGTTTTCAGCACC	21	61.4	179–159
USP18	Forward Primer	CCTGAGGCAAATCTGTCAGTC	21	60.4	21–41
	Reverse Primer	CGAACACCTGAATCAAGGAGTTA	23	60	220–198
GAPDH	Forward Primer	GGAGCGAGATCCCTCCAAAAT	21	61.6	108–128
	Reverse Primer	GGCTGTTGTCATACTTCTCATGG	23	60.9	304–282

**Table 2:** Basic information of healthy controls and COVID-19 patients.

	Healthy Controls	COVID-19	*p*
N	20	20	
Age	47.5, (32.75, 60.25)	49.45, (29.5, 69)	0.736
WBC, 10^9^/L	5.98, (5.3, 6.75)	5.53, (4.15, 7.05)	0.304
NEU%	57.64, (53.08, 62.3)	64.29, (56.83, 73.28)	0.012
LYM%	32.08, (27.58, 37.4)	22.08, (13.05, 32.1)	0.001
MONO%	6.86, (6.23, 7.6)	11.93, (8.73, 14.63)	<0.001
EOS%	2.86, (1.53, 4)	1.22, (0.23, 1.38)	<0.001
BASO%	0.71, (0.5, 0.98)	0.49, (0.33, 0.6)	0.010
NEU, 10^9^/L	3.56, (3.05, 3.7)	3.64, (2.6, 4.67)	0.790
LYM, 10^9^/L	2.06, (1.65, 2.48)	1.16, (0.69, 1.54)	<0.001
MONO, 10^9^/L	0.44, (0.33, 0.5)	0.65, (0.42, 0.87)	0.006
EOS, 10^9^/L	0.18, (0.15, 0.22)	0.06, (0.01, 0.1)	<0.001
BASO, 10^9^/L	0.09, (0.06, 0.12)	0.03, (0.02, 0.03)	<0.001
RBC, 10^12^/L	4.78, (4.6, 4.9)	4.38, (3.98, 4.67)	0.001
HGB, g/L	139.35, (124.25, 149)	125.35, (118.25, 136.75)	0.012
HCT%	0.42, (0.4, 0.44)	0.4, (0.37, 0.43)	0.025
MCV, fL	87.3, (85.23, 90.68)	91.46, (89.85, 94.9)	<0.001
MCH, Pg	29.93, (28.03, 31.58)	29.4, (28.7, 31)	0.838
MCHC, g/L	341.55, (332.25, 349.75)	321.08, (315.25, 327)	<0.001
RDW-SD, fL	42.55, (39, 45.75)	43.65, (41.1, 46)	0.293
RDW-CV	12.92, (12.53, 13.35)	13.16, (12.23, 13.68)	0.621
PLT, 10^9^/L	244.2, (132, 358.75)	171.8, (123.25, 219.25)	0.010
MPV, fL	8.56, (7.6, 9.5)	11.25, (10, 12.6)	<0.001
PDW	20.6, (18, 24.25)	14.12, (10.58, 16.8)	<0.001
PCT	0.26, (0.2, 0.32)	0.19, (0.15, 0.22)	<0.001
P-LCR	24.25, (16.25, 29)	34.57, (22.93, 46.13)	0.006
CRP	0.27(0.1, 0.4)	17.1, (1.97, 14.16)	<0.001

WBC: White blood cell; NEU: Neutrophil; LYM: Lymphocyte; MONO: Monocyte; BASO: Basophil; EOS: Eosinophils; NRBC: Nucleated red blood cell; HGB: Hemoglobin; HCT: Hematocrit; MCV: Erythrocyte mean corpuscular volume; MCH: Mean corpuscular hemoglobin; MCHC: Mean corpuscular hemoglobin concentration; RDW-SD: Red blood cell distribution width-standard deviation; RDW-CV: Red blood cell distribution width-coefficient of variance; PLT: Blood platelet; MPV: Mean platelet volume; PCT: Plateletcrit; PLCR: Platelet large cell ratio; CRP: C-reactive protein; PDW: Platelet distribution width.

## Data Availability

The original contributions presented in the study are included in the article/Supplementary Material, further inquiries can be directed to the corresponding authors.

## Supplementary Materials

The following supporting information can be downloaded at: https://www.mdpi.com/article/10.3390/microorganisms11082101/s1.
